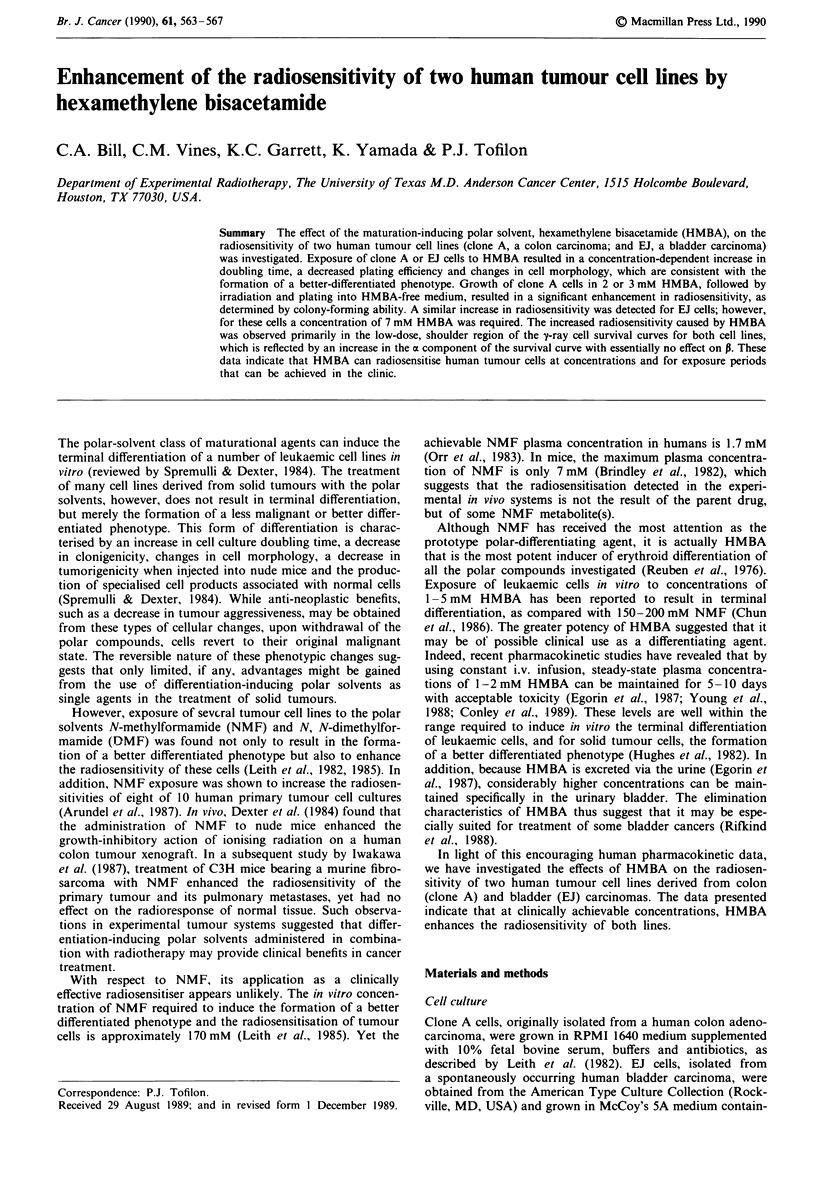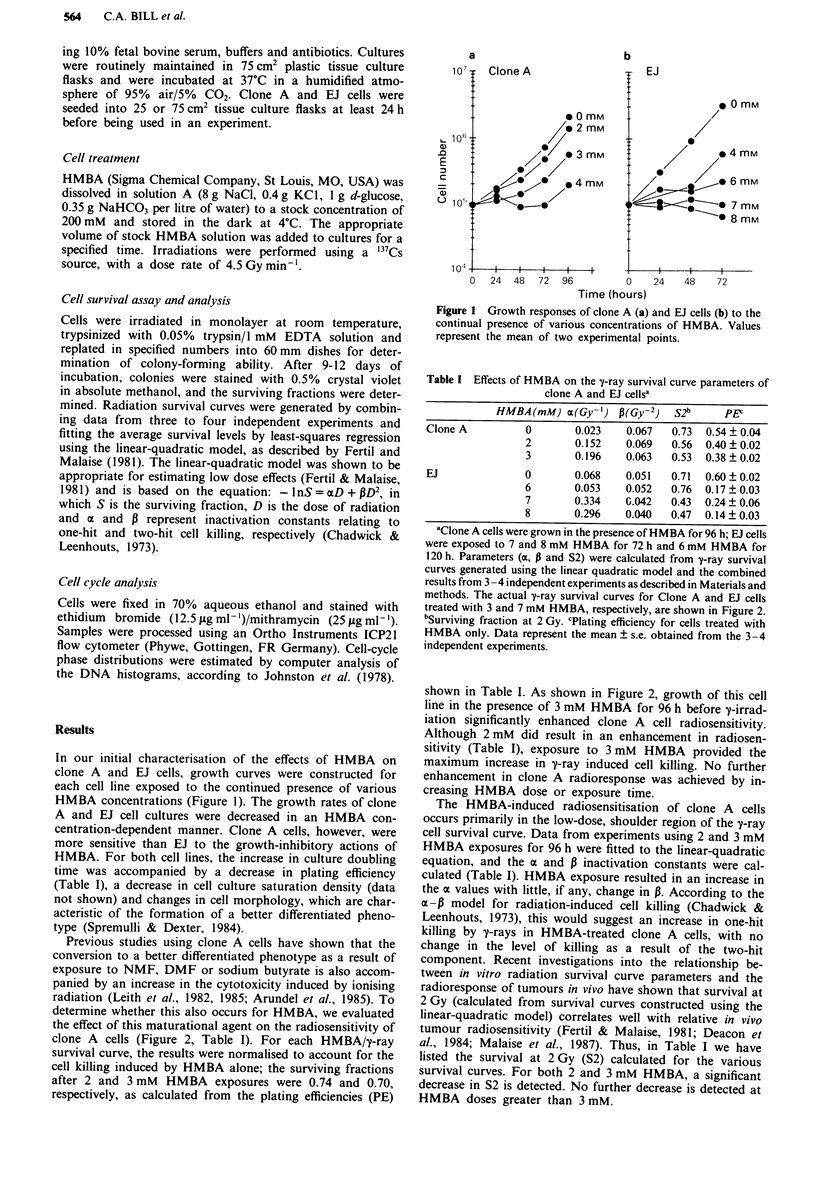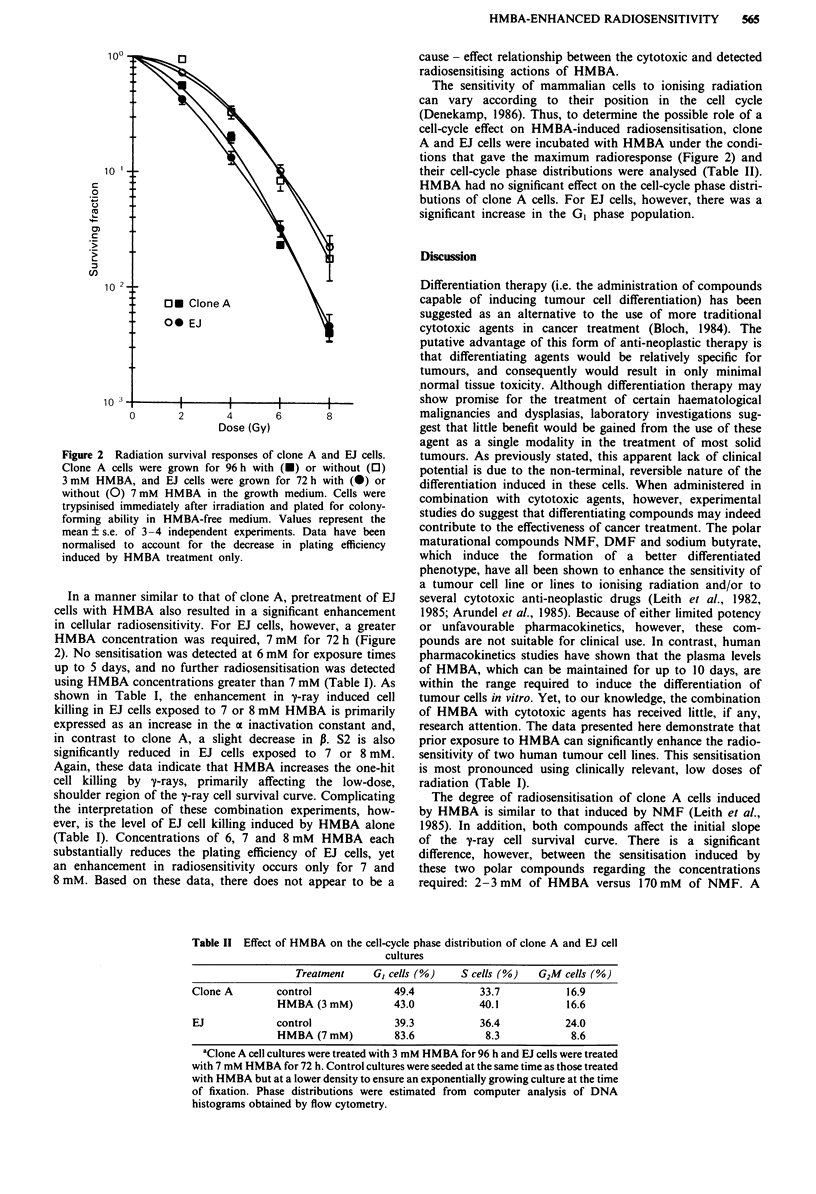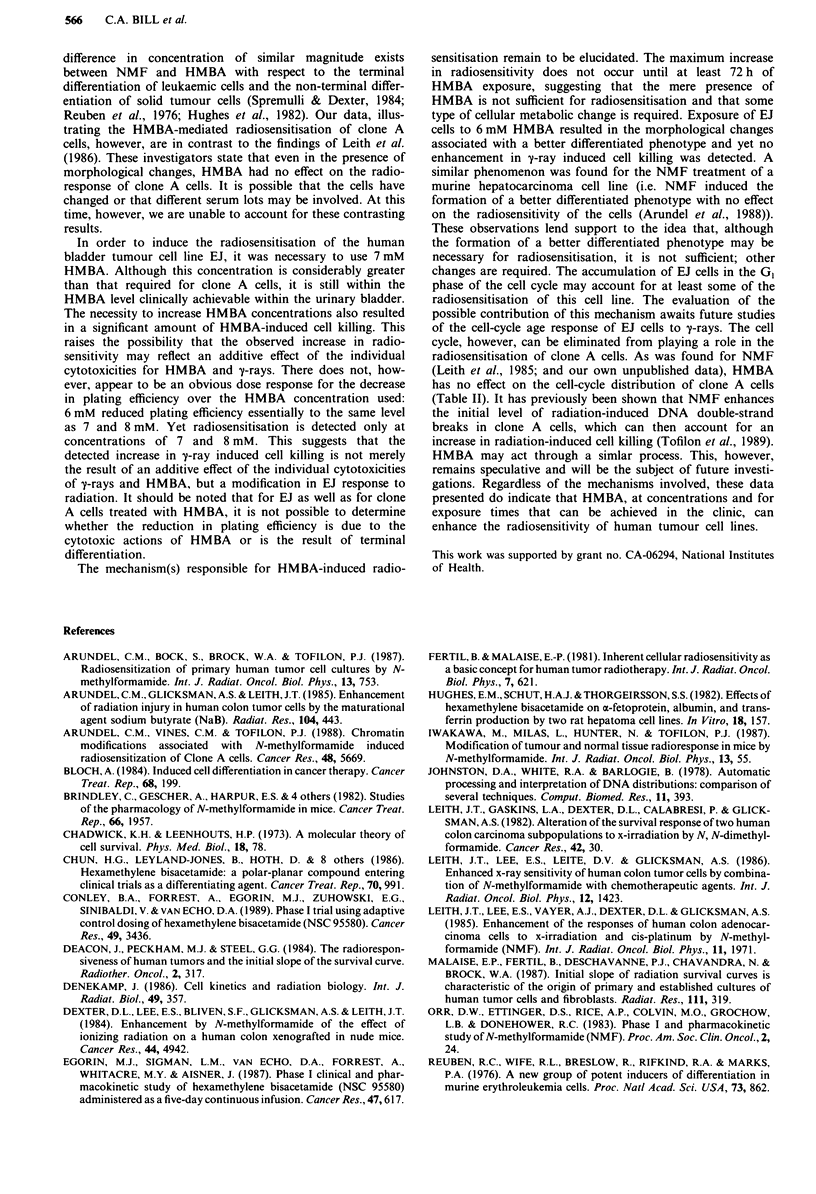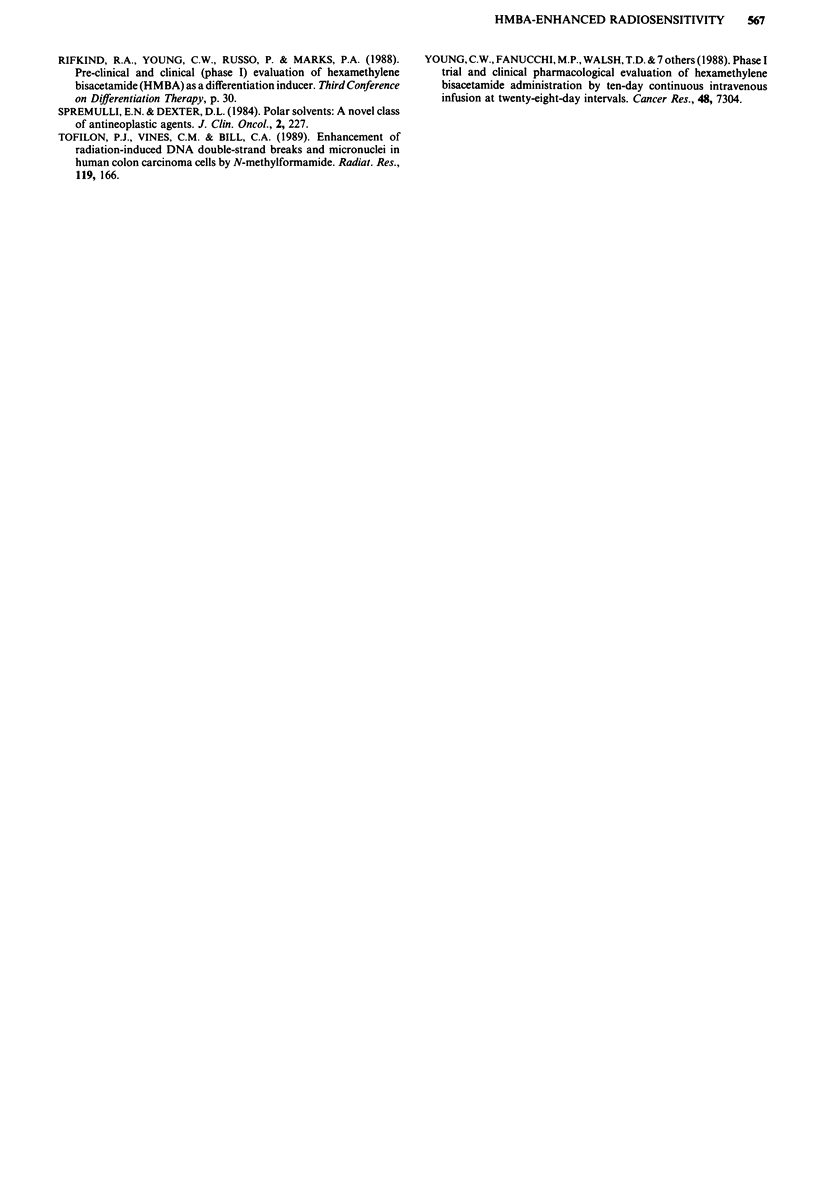# Enhancement of the radiosensitivity of two human tumour cell lines by hexamethylene bisacetamide.

**DOI:** 10.1038/bjc.1990.126

**Published:** 1990-04

**Authors:** C. A. Bill, C. M. Vines, K. C. Garrett, K. Yamada, P. J. Tofilon

**Affiliations:** Department of Experimental Radiotherapy, University of Texas M.D. Anderson Cancer Center, Houston 77030.

## Abstract

The effect of the maturation-inducing polar solvent, hexamethylene bisacetamide (HMBA), on the radiosensitivity of two human tumour cell lines (clone A, a colon carcinoma; and EJ, a bladder carcinoma) was investigated. Exposure of clone A or EJ cells to HMBA resulted in a concentration-dependent increase in doubling time, a decreased plating efficiency and changes in cell morphology, which are consistent with the formation of a better-differentiated phenotype. Growth of clone A cells in 2 or 3 mM HMBA, followed by irradiation and plating into HMBA-free medium, resulted in a significant enhancement in radiosensitivity, as determined by colony-forming ability. A similar increase in radiosensitivity was detected for EJ cells; however, for these cells a concentration of 7 mM HMBA was required. The increased radiosensitivity caused by HMBA was observed primarily in the low-dose, shoulder region of the gamma-ray cell survival curves for both cell lines, which is reflected by an increase in the alpha component of the survival curve with essentially no effect on beta. These data indicate that HMBA can radiosensitise human tumour cells at concentrations and for exposure periods that can be achieved in the clinic.


Br. J. Cancer (1990), 61, 563-567 © Macmillan Press Ltd., 1990

### Enhancement of the radiosensitivity of two human tumour cell lines by hexamethylene bisacetamide

C.A. Bill, C.M. Vines, K.C. Garrett, K. Yamada & P.J. Tofilon

*Department of Experimental Radiotherapy, The University of Texas M.D. Anderson Cancer Center, 1515 Holcombe Boulevard, Houston, TX 77030, USA.*

**Summary** The effect of the maturation-inducing polar solvent, hexamethylene bisacetamide (HMBA), on the radiosensitivity of two human tumour cell lines (clone A, a colon carcinoma; and EJ, a bladder carcinoma) was investigated. Exposure of clone A or EJ cells to HMBA resulted in a concentration-dependent increase in doubling time, a decreased plating efficiency and changes in cell morphology, which are consistent with the formation of a better-differentiated phenotype. Growth of clone A cells in 2 or 3 mM HMBA, followed by irradiation and plating into HMBA-free medium, resulted in a significant enhancement in radiosensitivity, as determined by colony-forming ability. A similar increase in radiosensitivity was detected for EJ cells; however, for these cells a concentration of 7 mM HMBA was required. The increased radiosensitivity caused by HMBA was observed primarily in the low-dose, shoulder region of the γ-ray cell survival curves for both cell lines, which is reflected by an increase in the α component of the survival curve with essentially no effect on β. These data indicate that HMBA can radiosensitise human tumour cells at concentrations and for exposure periods that can be achieved in the clinic.

The polar-solvent class of maturational agents can induce the terminal differentiation of a number of leukaemic cell lines *in vitro* (reviewed by Spremulli & Dexter, 1984). The treatment of many cell lines derived from solid tumours with the polar solvents, however, does not result in terminal differentiation, but merely the formation of a less malignant or better differentiated phenotype. This form of differentiation is characterised by an increase in cell culture doubling time, a decrease in clonigenicity, changes in cell morphology, a decrease in tumorigenicity when injected into nude mice and the production of specialised cell products associated with normal cells (Spremulli & Dexter, 1984). While anti-neoplastic benefits, such as a decrease in tumour aggressiveness, may be obtained from these types of cellular changes, upon withdrawal of the polar compounds, cells revert to their original malignant state. The reversible nature of these phenotypic changes suggests that only limited, if any, advantages might be gained from the use of differentiation-inducing polar solvents as single agents in the treatment of solid tumours.

However, exposure of several tumour cell lines to the polar solvents *N*-methylformamide (NMF) and *N*, *N*-dimethylformamide (DMF) was found not only to result in the formation of a better differentiated phenotype but also to enhance the radiosensitivity of these cells (Leith *et al.*, 1982, 1985). In addition, NMF exposure was shown to increase the radiosensitivities of eight of 10 human primary tumour cell cultures (Arundel *et al.*, 1987). *In vivo*, Dexter *et al.* (1984) found that the administration of NMF to nude mice enhanced the growth-inhibitory action of ionising radiation on a human colon tumour xenograft. In a subsequent study by Iwakawa *et al.* (1987), treatment of C3H mice bearing a murine fibrosarcoma with NMF enhanced the radiosensitivity of the primary tumour and its pulmonary metastases, yet had no effect on the radioresponse of normal tissue. Such observations in experimental tumour systems suggested that differentiation-inducing polar solvents administered in combination with radiotherapy may provide clinical benefits in cancer treatment.

With respect to NMF, its application as a clinically effective radiosensitiser appears unlikely. The *in vitro* concentration of NMF required to induce the formation of a better differentiated phenotype and the radiosensitisation of tumour cells is approximately 170 mM (Leith *et al.*, 1985). Yet the achievable NMF plasma concentration in humans is 1.7 mM (Orr *et al.*, 1983). In mice, the maximum plasma concentration of NMF is only 7 mM (Brindley *et al.*, 1982), which suggests that the radiosensitisation detected in the experimental *in vivo* systems is not the result of the parent drug, but of some NMF metabolite(s).

Although NMF has received the most attention as the prototype polar-differentiating agent, it is actually HMBA that is the most potent inducer of erythroid differentiation of all the polar compounds investigated (Reuben *et al.*, 1976). Exposure of leukaemic cells *in vitro* to concentrations of 1-5 mM HMBA has been reported to result in terminal differentiation, as compared with 150-200 mM NMF (Chun *et al.*, 1986). The greater potency of HMBA suggested that it may be of possible clinical use as a differentiating agent. Indeed, recent pharmacokinetic studies have revealed that by using constant i.v. infusion, steady-state plasma concentrations of 1-2 mM HMBA can be maintained for 5-10 days with acceptable toxicity (Egorin *et al.*, 1987; Young *et al.*, 1988; Conley *et al.*, 1989). These levels are well within the range required to induce *in vitro* the terminal differentiation of leukaemic cells, and for solid tumour cells, the formation of a better differentiated phenotype (Hughes *et al.*, 1982). In addition, because HMBA is excreted via the urine (Egorin *et al.*, 1987), considerably higher concentrations can be maintained specifically in the urinary bladder. The elimination characteristics of HMBA thus suggest that it may be especially suited for treatment of some bladder cancers (Rifkind *et al.*, 1988).

In light of this encouraging human pharmacokinetic data, we have investigated the effects of HMBA on the radiosensitivity of two human tumour cell lines derived from colon (clone A) and bladder (EJ) carcinomas. The data presented indicate that at clinically achievable concentrations, HMBA enhances the radiosensitivity of both lines.

### Materials and methods

### *Cell culture*

Clone A cells, originally isolated from a human colon adenocarcinoma, were grown in RPMI 1640 medium supplemented with 10% fetal bovine serum, buffers and antibiotics, as described by Leith *et al.* (1982). EJ cells, isolated from a spontaneously occurring human bladder carcinoma, were obtained from the American Type Culture Collection (Rockville, MD, USA) and grown in McCoy's 5A medium contain-

Correspondence: P.J. Tofilon.
Received 29 August 1989; and in revised form 1 December 1989.

ing 10% fetal bovine serum, buffers and antibiotics. Cultures were routinely maintained in 75 $cm^2$ plastic tissue culture flasks and were incubated at 37°C in a humidified atmosphere of 95% air/5% $CO_2$. Clone A and EJ cells were seeded into 25 or 75 $cm^2$ tissue culture flasks at least 24 h before being used in an experiment.

### *Cell treatment*

HMBA (Sigma Chemical Company, St Louis, MO, USA) was dissolved in solution A (8 g NaCl, 0.4 g KCl, 1 g *d*-glucose, 0.35 g $NaHCO_3$ per litre of water) to a stock concentration of 200 mM and stored in the dark at 4°C. The appropriate volume of stock HMBA solution was added to cultures for a specified time. Irradiations were performed using a $^{137}Cs$ source, with a dose rate of 4.5 Gy $min^{-1}$.

### *Cell survival assay and analysis*

Cells were irradiated in monolayer at room temperature, trypsinized with 0.05% trypsin/1 mM EDTA solution and replated in specified numbers into 60 mm dishes for determination of colony-forming ability. After 9-12 days of incubation, colonies were stained with 0.5% crystal violet in absolute methanol, and the surviving fractions were determined. Radiation survival curves were generated by combining data from three to four independent experiments and fitting the average survival levels by least-squares regression using the linear-quadratic model, as described by Fertil and Malaise (1981). The linear-quadratic model was shown to be appropriate for estimating low dose effects (Fertil & Malaise, 1981) and is based on the equation: $-\ln S = \alpha D + \beta D^2$, in which $S$ is the surviving fraction, $D$ is the dose of radiation and $\alpha$ and $\beta$ represent inactivation constants relating to one-hit and two-hit cell killing, respectively (Chadwick & Leenhouts, 1973).

### *Cell cycle analysis*

Cells were fixed in 70% aqueous ethanol and stained with ethidium bromide (12.5 $\mu g\ ml^{-1}$)/mithramycin (25 $\mu g\ ml^{-1}$). Samples were processed using an Ortho Instruments ICP21 flow cytometer (Phywe, Gottingen, FR Germany). Cell-cycle phase distributions were estimated by computer analysis of the DNA histograms, according to Johnston *et al.* (1978).

### Results

In our initial characterisation of the effects of HMBA on clone A and EJ cells, growth curves were constructed for each cell line exposed to the continued presence of various HMBA concentrations (Figure 1). The growth rates of clone A and EJ cell cultures were decreased in an HMBA concentration-dependent manner. Clone A cells, however, were more sensitive than EJ to the growth-inhibitory actions of HMBA. For both cell lines, the increase in culture doubling time was accompanied by a decrease in plating efficiency (Table I), a decrease in cell culture saturation density (data not shown) and changes in cell morphology, which are characteristic of the formation of a better differentiated phenotype (Spremulli & Dexter, 1984).

Previous studies using clone A cells have shown that the conversion to a better differentiated phenotype as a result of exposure to NMF, DMF or sodium butyrate is also accompanied by an increase in the cytotoxicity induced by ionising radiation (Leith *et al.*, 1982, 1985; Arundel *et al.*, 1985). To determine whether this also occurs for HMBA, we evaluated the effect of this maturational agent on the radiosensitivity of clone A cells (Figure 2, Table I). For each HMBA/γ-ray survival curve, the results were normalised to account for the cell killing induced by HMBA alone; the surviving fractions after 2 and 3 mM HMBA exposures were 0.74 and 0.70, respectively, as calculated from the plating efficiencies (PE)

**Figure 1** Growth responses of clone A (**a**) and EJ cells (**b**) to the continual presence of various concentrations of HMBA. Values represent the mean of two experimental points.

**Table I** Effects of HMBA on the γ-ray survival curve parameters of clone A and EJ cells[a]

| | *HMBA(mM)* | *α(Gy⁻¹)* | *β(Gy⁻²)* | *S2*[b] | *PE*[c] |
|---|---|---|---|---|---|
| Clone A | 0 | 0.023 | 0.067 | 0.73 | 0.54 ± 0.04 |
| | 2 | 0.152 | 0.069 | 0.56 | 0.40 ± 0.02 |
| | 3 | 0.196 | 0.063 | 0.53 | 0.38 ± 0.02 |
| EJ | 0 | 0.068 | 0.051 | 0.71 | 0.60 ± 0.02 |
| | 6 | 0.053 | 0.052 | 0.76 | 0.17 ± 0.03 |
| | 7 | 0.334 | C.042 | 0.43 | 0.24 ± 0.06 |
| | 8 | 0.296 | 0.040 | 0.47 | 0.14 ± 0.03 |

[a]Clone A cells were grown in the presence of HMBA for 96 h; EJ cells were exposed to 7 and 8 mM HMBA for 72 h and 6 mM HMBA for 120 h. Parameters (α, β and S2) were calculated from γ-ray survival curves generated using the linear quadratic model and the combined results from 3–4 independent experiments as described in Materials and methods. The actual γ-ray survival curves for Clone A and EJ cells treated with 3 and 7 mM HMBA, respectively, are shown in Figure 2. [b]Surviving fraction at 2 Gy. [c]Plating efficiency for cells treated with HMBA only. Data represent the mean ± s.e. obtained from the 3–4 independent experiments.

shown in Table I. As shown in Figure 2, growth of this cell line in the presence of 3 mM HMBA for 96 h before γ-irradiation significantly enhanced clone A cell radiosensitivity. Although 2 mM did result in an enhancement in radiosensitivity (Table I), exposure to 3 mM HMBA provided the maximum increase in γ-ray induced cell killing. No further enhancement in clone A radioresponse was achieved by increasing HMBA dose or exposure time.

The HMBA-induced radiosensitisation of clone A cells occurs primarily in the low-dose, shoulder region of the γ-ray cell survival curve. Data from experiments using 2 and 3 mM HMBA exposures for 96 h were fitted to the linear-quadratic equation, and the α and β inactivation constants were calculated (Table I). HMBA exposure resulted in an increase in the α values with little, if any, change in β. According to the α–β model for radiation-induced cell killing (Chadwick & Leenhouts, 1973), this would suggest an increase in one-hit killing by γ-rays in HMBA-treated clone A cells, with no change in the level of killing as a result of the two-hit component. Recent investigations into the relationship between *in vitro* radiation survival curve parameters and the radioresponse of tumours *in vivo* have shown that survival at 2 Gy (calculated from survival curves constructed using the linear-quadratic model) correlates well with relative *in vivo* tumour radiosensitivity (Fertil & Malaise, 1981; Deacon *et al.*, 1984; Malaise *et al.*, 1987). Thus, in Table I we have listed the survival at 2 Gy (S2) calculated for the various survival curves. For both 2 and 3 mM HMBA, a significant decrease in S2 is detected. No further decrease is detected at HMBA doses greater than 3 mM.

**Figure 2** Radiation survival responses of clone A and EJ cells. Clone A cells were grown for 96 h with (■) or without (□) 3 mM HMBA, and EJ cells were grown for 72 h with (●) or without (○) 7 mM HMBA in the growth medium. Cells were trypsinised immediately after irradiation and plated for colony-forming ability in HMBA-free medium. Values represent the mean ± s.e. of 3–4 independent experiments. Data have been normalised to account for the decrease in plating efficiency induced by HMBA treatment only.

In a manner similar to that of clone A, pretreatment of EJ cells with HMBA also resulted in a significant enhancement in cellular radiosensitivity. For EJ cells, however, a greater HMBA concentration was required, 7 mM for 72 h (Figure 2). No sensitisation was detected at 6 mM for exposure times up to 5 days, and no further radiosensitisation was detected using HMBA concentrations greater than 7 mM (Table I). As shown in Table I, the enhancement in γ-ray induced cell killing in EJ cells exposed to 7 or 8 mM HMBA is primarily expressed as an increase in the $\alpha$ inactivation constant and, in contrast to clone A, a slight decrease in $\beta$. S2 is also significantly reduced in EJ cells exposed to 7 or 8 mM. Again, these data indicate that HMBA increases the one-hit cell killing by γ-rays, primarily affecting the low-dose, shoulder region of the γ-ray cell survival curve. Complicating the interpretation of these combination experiments, however, is the level of EJ cell killing induced by HMBA alone (Table I). Concentrations of 6, 7 and 8 mM HMBA each substantially reduces the plating efficiency of EJ cells, yet an enhancement in radiosensitivity occurs only for 7 and 8 mM. Based on these data, there does not appear to be a cause – effect relationship between the cytotoxic and detected radiosensitising actions of HMBA.

The sensitivity of mammalian cells to ionising radiation can vary according to their position in the cell cycle (Denekamp, 1986). Thus, to determine the possible role of a cell-cycle effect on HMBA-induced radiosensitisation, clone A and EJ cells were incubated with HMBA under the conditions that gave the maximum radioresponse (Figure 2) and their cell-cycle phase distributions were analysed (Table II). HMBA had no significant effect on the cell-cycle phase distributions of clone A cells. For EJ cells, however, there was a significant increase in the $G_1$ phase population.

### Discussion

Differentiation therapy (i.e. the administration of compounds capable of inducing tumour cell differentiation) has been suggested as an alternative to the use of more traditional cytotoxic agents in cancer treatment (Bloch, 1984). The putative advantage of this form of anti-neoplastic therapy is that differentiating agents would be relatively specific for tumours, and consequently would result in only minimal normal tissue toxicity. Although differentiation therapy may show promise for the treatment of certain haematological malignancies and dysplasias, laboratory investigations suggest that little benefit would be gained from the use of these agent as a single modality in the treatment of most solid tumours. As previously stated, this apparent lack of clinical potential is due to the non-terminal, reversible nature of the differentiation induced in these cells. When administered in combination with cytotoxic agents, however, experimental studies do suggest that differentiating compounds may indeed contribute to the effectiveness of cancer treatment. The polar maturational compounds NMF, DMF and sodium butyrate, which induce the formation of a better differentiated phenotype, have all been shown to enhance the sensitivity of a tumour cell line or lines to ionising radiation and/or to several cytotoxic anti-neoplastic drugs (Leith *et al.*, 1982, 1985; Arundel *et al.*, 1985). Because of either limited potency or unfavourable pharmacokinetics, however, these compounds are not suitable for clinical use. In contrast, human pharmacokinetics studies have shown that the plasma levels of HMBA, which can be maintained for up to 10 days, are within the range required to induce the differentiation of tumour cells *in vitro*. Yet, to our knowledge, the combination of HMBA with cytotoxic agents has received little, if any, research attention. The data presented here demonstrate that prior exposure to HMBA can significantly enhance the radiosensitivity of two human tumour cell lines. This sensitisation is most pronounced using clinically relevant, low doses of radiation (Table I).

The degree of radiosensitisation of clone A cells induced by HMBA is similar to that induced by NMF (Leith *et al.*, 1985). In addition, both compounds affect the initial slope of the γ-ray cell survival curve. There is a significant difference, however, between the sensitisation induced by these two polar compounds regarding the concentrations required: 2–3 mM of HMBA versus 170 mM of NMF. A

**Table II** Effect of HMBA on the cell-cycle phase distribution of clone A and EJ cell cultures

| | Treatment | $G_1$ cells (%) | S cells (%) | $G_2M$ cells (%) |
|---|---|---|---|---|
| Clone A | control | 49.4 | 33.7 | 16.9 |
| | HMBA (3 mM) | 43.0 | 40.1 | 16.6 |
| EJ | control | 39.3 | 36.4 | 24.0 |
| | HMBA (7 mM) | 83.6 | 8.3 | 8.6 |

[a]Clone A cell cultures were treated with 3 mM HMBA for 96 h and EJ cells were treated with 7 mM HMBA for 72 h. Control cultures were seeded at the same time as those treated with HMBA but at a lower density to ensure an exponentially growing culture at the time of fixation. Phase distributions were estimated from computer analysis of DNA histograms obtained by flow cytometry.

difference in concentration of similar magnitude exists between NMF and HMBA with respect to the terminal differentiation of leukaemic cells and the non-terminal differentiation of solid tumour cells (Spremulli & Dexter, 1984; Reuben *et al.*, 1976; Hughes *et al.*, 1982). Our data, illustrating the HMBA-mediated radiosensitisation of clone A cells, however, are in contrast to the findings of Leith *et al.* (1986). These investigators state that even in the presence of morphological changes, HMBA had no effect on the radioresponse of clone A cells. It is possible that the cells have changed or that different serum lots may be involved. At this time, however, we are unable to account for these contrasting results.

In order to induce the radiosensitisation of the human bladder tumour cell line EJ, it was necessary to use 7 mM HMBA. Although this concentration is considerably greater than that required for clone A cells, it is still within the HMBA level clinically achievable within the urinary bladder. The necessity to increase HMBA concentrations also resulted in a significant amount of HMBA-induced cell killing. This raises the possibility that the observed increase in radiosensitivity may reflect an additive effect of the individual cytotoxicities for HMBA and γ-rays. There does not, however, appear to be an obvious dose response for the decrease in plating efficiency over the HMBA concentration used: 6 mM reduced plating efficiency essentially to the same level as 7 and 8 mM. Yet radiosensitisation is detected only at concentrations of 7 and 8 mM. This suggests that the detected increase in γ-ray induced cell killing is not merely the result of an additive effect of the individual cytotoxicities of γ-rays and HMBA, but a modification in EJ response to radiation. It should be noted that for EJ as well as for clone A cells treated with HMBA, it is not possible to determine whether the reduction in plating efficiency is due to the cytotoxic actions of HMBA or is the result of terminal differentiation.

The mechanism(s) responsible for HMBA-induced radiosensitisation remain to be elucidated. The maximum increase in radiosensitivity does not occur until at least 72 h of HMBA exposure, suggesting that the mere presence of HMBA is not sufficient for radiosensitisation and that some type of cellular metabolic change is required. Exposure of EJ cells to 6 mM HMBA resulted in the morphological changes associated with a better differentiated phenotype and yet no enhancement in γ-ray induced cell killing was detected. A similar phenomenon was found for the NMF treatment of a murine hepatocarcinoma cell line (i.e. NMF induced the formation of a better differentiated phenotype with no effect on the radiosensitivity of the cells (Arundel *et al.*, 1988)). These observations lend support to the idea that, although the formation of a better differentiated phenotype may be necessary for radiosensitisation, it is not sufficient; other changes are required. The accumulation of EJ cells in the $G_1$ phase of the cell cycle may account for at least some of the radiosensitisation of this cell line. The evaluation of the possible contribution of this mechanism awaits future studies of the cell-cycle age response of EJ cells to γ-rays. The cell cycle, however, can be eliminated from playing a role in the radiosensitisation of clone A cells. As was found for NMF (Leith *et al.*, 1985; and our own unpublished data), HMBA has no effect on the cell-cycle distribution of clone A cells (Table II). It has previously been shown that NMF enhances the initial level of radiation-induced DNA double-strand breaks in clone A cells, which can then account for an increase in radiation-induced cell killing (Tofilon *et al.*, 1989). HMBA may act through a simlar process. This, however, remains speculative and will be the subject of future investigations. Regardless of the mechanisms involved, these data presented do indicate that HMBA, at concentrations and for exposure times that can be achieved in the clinic, can enhance the radiosensitivity of human tumour cell lines.

This work was supported by grant no. CA-06294, National Institutes of Health.

### References

ARUNDEL, C.M., BOCK, S., BROCK, W.A. & TOFILON, P.J. (1987). Radiosensitization of primary human tumor cell cultures by *N*-methylformamide. *Int. J. Radiat. Oncol. Biol. Phys.*, **13**, 753.

ARUNDEL, C.M., GLICKSMAN, A.S. & LEITH, J.T. (1985). Enhancement of radiation injury in human colon tumor cells by the maturational agent sodium butyrate (NaB). *Radiat. Res.*, **104**, 443.

ARUNDEL, C.M., VINES, C.M. & TOFILON, P.J. (1988). Chromatin modifications associated with *N*-methylformamide induced radiosensitization of Clone A cells. *Cancer Res.*, **48**, 5669.

BLOCH, A. (1984). Induced cell differentiation in cancer therapy. *Cancer Treat. Rep.*, **68**, 199.

BRINDLEY, C., GESCHER, A., HARPUR, E.S. & 4 others (1982). Studies of the pharmacology of *N*-methylformamide in mice. *Cancer Treat. Rep.*, **66**, 1957.

CHADWICK, K.H. & LEENHOUTS, H.P. (1973). A molecular theory of cell survival. *Phys. Med. Biol.*, **18**, 78.

CHUN, H.G., LEYLAND-JONES, B., HOTH, D. & 8 others (1986). Hexamethylene bisacetamide: a polar-planar compound entering clinical trials as a differentiating agent. *Cancer Treat. Rep.*, **70**, 991.

CONLEY, B.A., FORREST, A., EGORIN, M.J., ZUHOWSKI, E.G., SINIBALDI, V. & VAN ECHO, D.A. (1989). Phase I trial using adaptive control dosing of hexamethylene bisacetamide (NSC 95580). *Cancer Res.*, **49**, 3436.

DEACON, J., PECKHAM, M.J. & STEEL, G.G. (1984). The radioresponsiveness of human tumors and the initial slope of the survival curve. *Radiother. Oncol.*, **2**, 317.

DENEKAMP, J. (1986). Cell kinetics and radiation biology. *Int. J. Radiat. Biol.*, **49**, 357.

DEXTER, D.L., LEE, E.S., BLIVEN, S.F., GLICKSMAN, A.S. & LEITH, J.T. (1984). Enhancement by *N*-methylformamide of the effect of ionizing radiation on a human colon xenografted in nude mice. *Cancer Res.*, **44**, 4942.

EGORIN, M.J., SIGMAN, L.M., VAN ECHO, D.A., FORREST, A., WHITACRE, M.Y. & AISNER, J. (1987). Phase I clinical and pharmacokinetic study of hexamethylene bisacetamide (NSC 95580) administered as a five-day continuous infusion. *Cancer Res.*, **47**, 617.

FERTIL, B. & MALAISE, E.-P. (1981). Inherent cellular radiosensitivity as a basic concept for human tumor radiotherapy. *Int. J. Radiat. Oncol. Biol. Phys.*, **7**, 621.

HUGHES, E.M., SCHUT, H.A.J. & THORGEIRSSON, S.S. (1982). Effects of hexamethylene bisacetamide on α-fetoprotein, albumin, and transferrin production by two rat hepatoma cell lines. *In Vitro*, **18**, 157.

IWAKAWA, M., MILAS, L., HUNTER, N. & TOFILON, P.J. (1987). Modification of tumour and normal tissue radioresponse in mice by *N*-methylformamide. *Int. J. Radiat. Oncol. Biol. Phys.*, **13**, 55.

JOHNSTON, D.A., WHITE, R.A. & BARLOGIE, B. (1978). Automatic processing and interpretation of DNA distributions: comparison of several techniques. *Comput. Biomed. Res.*, **11**, 393.

LEITH, J.T., GASKINS, L.A., DEXTER, D.L., CALABRESI, P. & GLICKSMAN, A.S. (1982). Alteration of the survival response of two human colon carcinoma subpopulations to x-irradiation by *N*, *N*-dimethylformamide. *Cancer Res.*, **42**, 30.

LEITH, J.T., LEE, E.S., LEITE, D.V. & GLICKSMAN, A.S. (1986). Enhanced x-ray sensitivity of human colon tumor cells by combination of *N*-methylformamide with chemotherapeutic agents. *Int. J. Radiat. Oncol. Biol. Phys.*, **12**, 1423.

LEITH, J.T., LEE, E.S., VAYER, A.J., DEXTER, D.L. & GLICKSMAN, A.S. (1985). Enhancement of the responses of human colon adenocarcinoma cells to x-irradiation and cis-platinum by *N*-methylformamide (NMF). *Int. J. Radiat. Oncol. Biol. Phys.*, **11**, 1971.

MALAISE, E.P., FERTIL, B., DESCHAVANNE, P.J., CHAVANDRA, N. & BROCK, W.A. (1987). Initial slope of radiation survival curves is characteristic of the origin of primary and established cultures of human tumor cells and fibroblasts. *Radiat. Res.*, **111**, 319.

ORR, D.W., ETTINGER, D.S., RICE, A.P., COLVIN, M.O., GROCHOW, L.B. & DONEHOWER, R.C. (1983). Phase I and pharmacokinetic study of *N*-methylformamide (NMF). *Proc. Am. Soc. Clin. Oncol.*, **2**, 24.

REUBEN, R.C., WIFE, R.L., BRESLOW, R., RIFKIND, R.A. & MARKS, P.A. (1976). A new group of potent inducers of differentiation in murine erythroleukemia cells. *Proc. Natl Acad. Sci. USA*, **73**, 862.

RIFKIND, R.A., YOUNG, C.W., RUSSO, P. & MARKS, P.A. (1988). Pre-clinical and clinical (phase I) evaluation of hexamethylene bisacetamide (HMBA) as a differentiation inducer. *Third Conference on Differentiation Therapy*, p. 30.

SPREMULLI, E.N. & DEXTER, D.L. (1984). Polar solvents: A novel class of antineoplastic agents. *J. Clin. Oncol.*, **2**, 227.

TOFILON, P.J., VINES, C.M. & BILL, C.A. (1989). Enhancement of radiation-induced DNA double-strand breaks and micronuclei in human colon carcinoma cells by *N*-methylformamide. *Radiat. Res.*, **119**, 166.

YOUNG, C.W., FANUCCHI, M.P., WALSH, T.D. & 7 others (1988). Phase I trial and clinical pharmacological evaluation of hexamethylene bisacetamide administration by ten-day continuous intravenous infusion at twenty-eight-day intervals. *Cancer Res.*, **48**, 7304.